# Recent advances in enzyme-related biomaterials for arthritis treatment

**DOI:** 10.3389/fchem.2022.988051

**Published:** 2022-08-16

**Authors:** Xin-Hao Liu, Jia-Ying Ding, Zhi-Heng Zhu, Xi-Chen Wu, Yong-Jia Song, Xiao-Ling Xu, Dao-Fang Ding

**Affiliations:** ^1^ Center of Rehabilitation Medicine, Yueyang Hospital, Shanghai University of Traditional Chinese Medicine, Shanghai, China; ^2^ School of Rehabilitation Science, Shanghai University of Traditional Chinese Medicine, Shanghai, China; ^3^ Shulan International Medical College, Zhejiang Shuren University, Hangzhou, China

**Keywords:** biomaterials, nano-therapy, arthritis, matrix metalloproteinases, endogenous antioxidant enzymes

## Abstract

Arthritis is a group of highly prevalent joint disorders, and osteoarthritis (OA) and rheumatoid arthritis are the two most common types. The high prevalence of arthritis causes severe burdens on individuals, society and the economy. Currently, the primary treatment of arthritis is to relieve symptoms, but the development of arthritis cannot be effectively prevented. Studies have revealed that the disrupted balance of enzymes determines the pathological changes in arthritis. In particular, the increased levels of matrix metalloproteinases and the decreased expression of endogenous antioxidant enzymes promote the progression of arthritis. New therapeutic strategies have been developed based on the expression characteristics of these enzymes. Biomaterials have been designed that are responsive when the destructive enzymes MMPs are increased or have the activities of the antioxidant enzymes that play a protective role in arthritis. Here, we summarize recent studies on biomaterials associated with MMPs and antioxidant enzymes involved in the pathological process of arthritis. These enzyme-related biomaterials have been shown to be beneficial for arthritis treatment, but there are still some problems that need to be solved to improve efficacy, especially penetrating the deeper layer of articular cartilage and targeting osteoclasts in subchondral bone. In conclusion, enzyme-related nano-therapy is challenging and promising for arthritis treatment.

## Introduction

Arthritis, which is a group of musculoskeletal diseases, is one of the leading causes of disability in the elderly population (Woolf and Pfleger, 2003). Osteoarthritis (OA) and rheumatoid arthritis (RA) are the most prevalent types of arthritis and affected 344 million people and 13 million people, respectively, globally in 2019 (Cieza et al., 2021). OA is characterized by joint degeneration, especially in the knee, and involves multiple joints, such as the hand, hip, knee and foot. A large-scale survey in the United Kingdom in 2017 showed that the prevalence of OA in adults was 10.7% (Swain et al., 2020-06). The increases in obesity and the ageing population contribute to the prevalence of OA (Briggs et al., 2020-10). RA is an immunization-induced systemic disease characterized by synovial inflammation and joint destruction, and the prevalence of RA is 0.5–1.0% in the US (Palmer et al., 2019).

OA and RA are both inflammatory joint diseases that involve joint and synovial destruction and immune cell infiltration (Zhang et al., 2019-03) and is associated with joint pain, swelling, and limited movement, resulting in a decline in physical function, increased dependence and reduced quality of life. Furthermore, the prevalence of OA and RA is expected to increase significantly as the global population ages. The treatment of arthritis is often a long and complex process due to irreversible damage and the risk of comorbidities, resulting in extremely high medical and economic burdens on society, and these burdens continue to increase globally (Briggs et al., 2020-10).

To date, there is no effective cure for OA or RA. The current interventions include medications, physical therapy, and surgical intervention, all of which are aimed at alleviating symptoms and reducing joint damage and disability. Medications for OA, including topical, oral and intra-articular (IA) injectable drugs, are palliative and limited to controlling symptoms of joint swelling, pain and stiffness (Tschon et al., 2020-06). A randomized clinical trial has even shown that IA corticosteroids may accelerate the destruction of articular cartilage (McAlindon et al., 2017-05). Currently, non-steroidal anti-inflammatory drugs (NSAIDs), glucocorticoids (GCs) and disease-modifying anti-rheumatic drugs (DMARDs) are mainly used in the clinical treatment of RA. The targets of traditional DMARDs are not clear, and approximately 30%–50% of patients respond poorly to these drugs (Sparks, 2019). As a result of the poor bioavailability and short half-lives of anti-rheumatic drugs, prolonged repeated use can cause serious adverse reactions such as vomiting, drug resistance and bone marrow suppression.

Physical therapy for OA and RA includes weight loss, moderate exercise and knee joint distraction. Knee joint distraction can improve symptoms and promote tissue repair in severe knee joint degeneration, but there is frequent infection during the follow-up (Jansen and Mastbergen, 2022-01; van der Woude et al., 2017-01). When conservative treatment is not feasible for end-stage arthritis, surgical intervention, such as total joint replacement, can be considered, but this treatment strategy is related to persistent postsurgical pain and infection (Wylde et al., 2011-03; Chung et al., 2021-11).

Currently, new therapeutic strategies and drugs primarily alleviate symptoms to treat arthritis, and critically unsolved problems, such as how to restore abnormal cellular function in arthritis, should be considered. Cellular activity depends on various proteins, and some of these proteins are important enzymes for physiological and pathological processes. Herein, we summarized the essential enzymes that are involved in pathological changes in arthritis.

### Arthritis-related enzymes

The pathological changes in OA and RA are mainly characterized by cartilage destruction and synovial inflammation (Trachana et al., 2019; Scherer et al., 2020). Cell metabolism is often regulated by different enzymes, and abnormal levels of enzymes are typically associated with the occurrence of various diseases. In cartilage, different matrix metalloproteinases (MMPs) are responsible for destroying chondrocytes by degrading collagen and proteoglycans.

#### 1 matrix metalloproteinases linked with arthritis

There is increasing evidence that these inflammatory mediators are involved in the pathogenesis of both OA and RA (Malemud, 2017; van Dalen et al., 2017). Neutrophils, monocytes and macrophages infiltrate cartilage and synovial tissue after inflammation occurs, releasing various inflammatory factors and chemokines, which cause an increase in MMPs.

The destruction or degradation of articular cartilage is regulated by MMPs, which are a family of proteolytic enzymes that hydrolyse extracellular matrix (ECM). Different types of MMPs are involved in degrading proteoglycans and collagens, which are the main components of ECM in cartilage, especially MMP-1, MMP-2, MMP-3, MMP-9 and MMP-13 (Itoh, 2017; Mehana et al., 2019). MMPs can degrade collagen, elastin, and other substances in the ECM of articular cartilage that maintain the structure of cartilage and ultimately destroy the integrity of ECM structure and function.

Under pathological conditions, the expression level of MMP-1 was significantly increased in OA and RA, and this factor degraded ECM collagen and mediated cartilage destruction (Wang et al., 2020a). In cartilage and synovium, MMP-1 expression increased steadily during the progression of OA in a rabbit model of anterior cruciate ligament transection (ACLT) (Wu et al., 2008). MMP-1 could lead to the degeneration of primary collagen (type Ⅱ collagen) in cartilage, and this effect was irreversible (Macdonald et al., 2018).

The development of OA and RA is associated with the increased secretion and activity of MMP-2 in synovial cells and the joints of RA patients, respectively (Kim et al., 2011; Galasso et al., 2012). Furthermore, MMP-2-sensitive peptide was shown to be specifically released in inflammatory joints *in vitro* and *in vivo*, which might be an important approach for drug-targeted treatment of RA (Yu et al., 2022).

Significantly increased levels of MMP-3 in the serum of OA patients were positively correlated with the severity of knee OA and RA in patients (Ma et al., 2014; Georgiev et al., 2018; Pengas et al., 2018). Furthermore, serum MMP-3 levels were closely correlated with disease activity scores, suggesting that serum MMP-3 levels could be used as an indicator of structural damage and monitor disease progression (Galil et al., 2016; Tuncer et al., 2019).

MMP-9 was also positively correlated with disease severity in OA patients (Lipari and Gerbino, 2013). A meta-analysis showed that MMP-2 and MMP-9 protein expression levels were significantly higher in the OA group than in the control group, indicating that MMP-2 and MMP-9 are involved in the pathogenesis of OA (Zeng et al., 2015). Multiple studies have shown that the expression of MMP-9 in synovial fluid and synovial cells of RA patients is increased (Silosi et al., 2015; Ma et al., 2019). The degree of inflammation in RA patients correlated with Toll-like receptor 2 (TLR2) expression in peripheral blood monocytes. The increased expression of TLR2 led to the increased expression of MMP-9 (Chen et al., 2015). MMP-9 could participate in the synovial cell-mediated inflammatory response and the degeneration of ECM, especially proteoglycans, which might directly cause joint destruction (Metzger et al., 2012).

MMP-13 is a crucial enzyme leading to the degradation of collagen types I, II and III and the cartilage proteoglycan aggrecans and is considered a significant factor in the pathogenesis of OA (Fosang et al., 1996). MMP-13 attracted much attention due to its obvious overexpression in the articular cartilage of OA patients, but it was almost undetectable in normal adult tissues (Kaneva, 2022). Interfering with the expression of MMP-13 in a surgically induced OA model could efficiently alleviate OA severity (Hoshi et al., 2017). Given its critical role in ECM degradation, MMP-13 has been a promising target in OA treatment (Hu and Ecker, 2021). K/BxN serum-induced arthritis increases MMP-13 expression in C57BL/6 mice, and MMP-13-deficient (MMP-13−/−) mice exhibit reduced inflammation and joint destruction (Singh et al., 2013). In addition, MMP-13 was also associated with the progression of RA, providing crucial predictive information about future structural damage and severity in early RA patients (Tatematsu et al., 2018).

#### 2 Endogenous antioxidant enzymes linked with arthritis

Apart from the direct effect of MMPs on ECM degradation in cartilage and promoting the progression of arthritis, endogenous antioxidants such as superoxide dismutases (SODs), glutathione peroxidase (GPx), catalase (CAT), and glutathione reductase (GR) also affect the occurrence of arthritis by scavenging intracellular reactive oxygen species (ROS) and alleviating cellular oxidative stress.

ROS are key signalling molecules in the progression of inflammatory diseases (Mittal et al., 2014). Under inflammatory conditions, the oxidative stress induced by macrophages, monocytes, and neutrophils leads to the formation of interendothelial junctions, accelerating the crossing of the endothelial barrier and ultimately promoting inflammation (You et al., 2018).

The levels of intra-articular ROS (including H_2_O_2_, O_2_^−^, OH^−^, and HOCl) are significantly increased in OA patients, while ROS are maintained at low levels in normal articular tissue (Lepetsos and Papavassiliou, 2016; Yao et al., 2019). The overproduction of ROS causes overoxidation, protein carbonylation, and DNA damage and is considered the primary mechanism of chondrocyte loss and tissue damage (Hosseinzadeh et al., 2016). The associated ROS, including nitric oxide (NO), superoxide anion (O_2_^−^) and hydrogen peroxide (H_2_O_2_), are present in the articular cavities of RA patients in large quantities (Datta et al., 2014). When the local inflammatory response in RA joints is accelerated and ROS levels exceed physiological tolerance, they not only damage proteins, lipids, and nucleic acids but also act as important endogenous signalling regulators that amplify the synovial inflammatory response (Bala et al., 2017; Phull et al., 2018). Li et al. found that ROS significantly promoted the proliferation of RA synovial fibroblasts and the production of inflammatory factors and that inhibiting ROS significantly downregulated the inflammatory factors secreted by RA synovial fibroblasts, ultimately improving RA conditions (Li et al., 2018). Therefore, a potent antioxidant compound that can reduce ROS in inflammatory cells may be a key factor in the treatment of chronic inflammatory diseases.

ROS clearance is regulated by SODs, GPx, CAT and GR (He et al., 2017). CAT and GPx are involved in the decomposition of intracellular hydrogen peroxide and maintain normal ROS levels to reduce toxic reactions. SOD can catalyse O_2_^−^ into O_2_ and H_2_O_2_. GR catalyses the reduction of glutathione disulfide (GSSG) to the sulfhydryl form of glutathione (GSH), which plays an important role in the tissue oxidative stress response (Deponte, 2013). The levels of SOD, CAT and other antioxidant enzymes in OA chondrocytes were significantly lower than those in normal chondrocytes, indicating that insufficient antioxidant capacity might cause cartilage damage (Zhuang et al., 2018). Unlike the expression pattern of other antioxidant enzymes, the expression of GR was increased in arthritis (Meshkibaf et al., 2019; Idzik et al., 2022).

The proliferation and activation of osteoclasts (OCs) are key factors leading to bone damage and bone metabolism disorders in RA (Auréal et al., 2020). Recent studies have shown a close correlation between bone destruction and oxidative stress in the pathogenesis of RA. ROS promote osteoclast differentiation (Gamal et al., 2018). Decreased expression of SOD, CAT and GPx was found in the ankle joints of RA rats (Ren et al., 2019a). ROS-induced peroxidation is inhibited by antioxidant enzymes, among which superoxide dismutase 3 (SOD3) is the key enzyme that protects cells from oxidative stress (Nguyen et al., 2020). SOD3 reduced proinflammatory cytokines (IL-1β, IL-2, IL-4, and TNF-α) and the release of MMPs (MMP-2, MMP-3 and MMP-9), ultimately inhibiting inflammatory responses (Xie et al., 2021). Icariin protects synoviocytes induced by lipopolysaccharide (LPS) by inhibiting ferroptosis by activating the Xc/GPX4 axis (Luo and Zhang, 2021).

Considering the importance of MMPs and oxide reductase associated with ROS in the occurrence of arthritis, biomaterials that target endogenous enzymes have become a hot research topic in recent years. Next, we will introduce the application of biomaterials that are linked with these enzymes.

### Nanotherapies that target enzymes in arthritis

**FIGURE 1:**
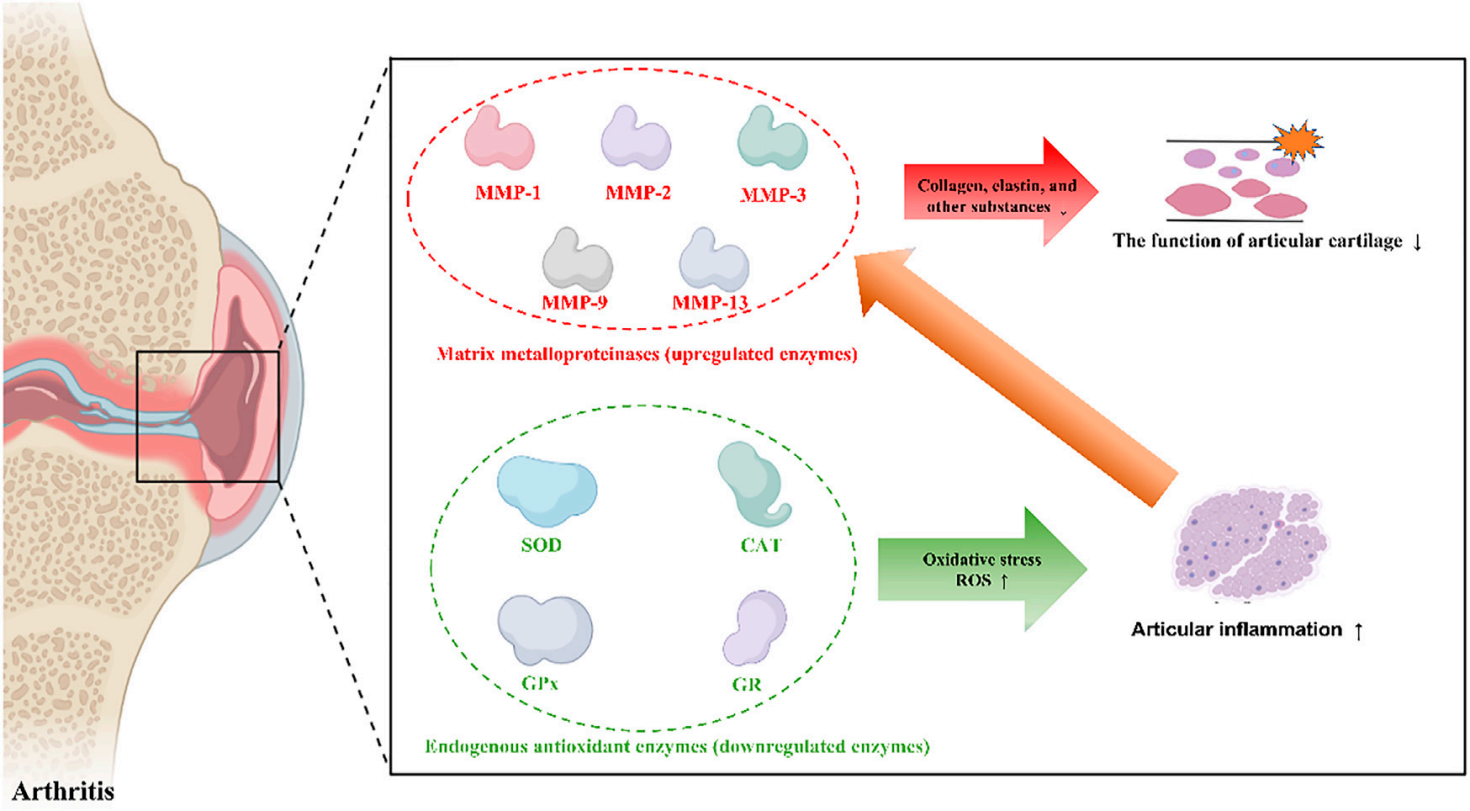
Upregulated enzymes (red) and downregulated enzymes (green) in arthritis pathology.

Enzymes that play critical roles in arthritis pathology are categorized into two groups according to their expression characteristics: upregulated enzymes and downregulated enzymes, which are listed in Figure 1. Enzyme homeostasis is critical for the human body. Both the upregulated and downregulated expression of these enzymes disrupt the balance of cell metabolism and can cause diseases. Therefore, therapeutic strategies have been designed according to the expression of these enzymes. If the expression of these enzymes is upregulated, nanomaterials can respond and release an effective drug to inhibit pathological changes, or nanomaterials can be fabricated to simulate the effects of downregulated enzymes. Next, we described two different functional enzymes in arthritis treatment.

#### 1 Nanomaterials associated with upregulated enzymes in arthritis treatments

It is well known that MMPs are the main destructive enzymes in chondrocytes that degrade ECM components, such as proteoglycans and collagen networks. Degradation of the ECM leads to functional destruction of chondrocytes and cartilage erosion. Therefore, MMPs have become an important molecular target for studies on the treatment of OA. In particular, MMP-13, a critical protease in chondrocytes, is responsible for the degradation of type II collagen and proteoglycans.

OA is a chronic inflammatory disease. Growing evidence reveals that the changes in the OA microenvironment include excessive inflammation and MMP overexpression (Latourte et al., 2017; Li et al., 2017; Stocco et al., 2019). The microenvironment is an important factor in maintaining joint homeostasis. Long-term inhibition of MMP enzymatic activity may lead to adverse reactions. Therefore, it is necessary to design materials that are highly selective for MMPs and can adapt to the changes in MMP levels *in vivo*. When MMP expression is upregulated, MMP-responsive nanoparticles (NPs) work, and they are inactive when MMPs are at low levels.

The increased expression of MMPs in inflamed tissues may be a promising breakthrough for arthritis therapy. A commercially available, Food and Drug Administration (FDA)-approved molecule known as triglycerol monostearate (TGMS) has been shown to be responsive to MMPs (Wen et al., 2019).

MMP-responsive PEGylated lipid NPs (TGMS/DSPE-PEG2000 NPs) can be produced through the coassembly of TGMS and 1,2-distearoyl-sn-glycero-3-phosphoethanolamine-poly (ethylene glycol) (DSPE-PEG2000). Dexamethasone (Dex)-loaded MMP-responsive NPs were obtained by loading TGMS/DSPE-PEG2000 NPs with Dex, and Dex can be rapidly released from the lipid NPs after TGMS is cleaved by MMP-3 and MMP-9. Dex-loaded MMP-responsive NPs significantly reduced the degree of joint swelling and inhibited the production of TNF-α and IL-1β in the joint (He et al., 2020).

In another study, the nanozyme-like role of the hydrogel form of TGMS(TG-18) was further confirmed in RA treatment. A hydrogel platform that exhibits disassembly and drug release controlled by the concentration of enzymes during arthritis flares was constructed. In this study, a triglycerol monostearate hydrogel (TG-18) loaded with the corticosteroid triamcinolone acetonide (TA) exhibited drug release in response to the increased activities of arthritis-related enzymes *in vitro* (MMP-2, MMP-3, MMP-9) or synovial fluid from patients with RA (Joshi et al., 2018).

In addition to synovial inflammation and joint swelling, obvious cartilage damage and bone erosion are often observed in RA. Synovial macrophages mediate joint inflammation once activated, and OCs are responsible for arthritic bone erosion and resorption of the bone matrix. Both OCs and synovial macrophages express high levels of αvβ3 integrin, which plays an important role in activated macrophage-dependent inflammation and OC-dependent bone resorption. Macrophages and OCs fail to undergo apoptosis in the RA joint, leading to persistent inflammation and joint destruction. Therefore, inducing OC and macrophage apoptosis in RA joints represents a promising strategy for advanced RA treatment. According to the characteristics of OCs and synovial macrophages, novel CEL-loaded PRNPs (CEL-PRNPs) were synthesized that contained celastrol (CEL), which can induce apoptosis in OCs and macrophages, RGD, which is a ligand of αvβ3 that targets OCs and inflammatory macrophages, and polyethylene glycol (PEG), which is cleaved by MMP-9. In an adjuvant-induced arthritis rat model, CEL-PRNPs efficiently reduced the number of OCs and inflammatory macrophages and relieved various symptoms, including ankle and paw swelling and bone erosion, in the inflamed joints of AIA rats with advanced arthritis (Deng et al., 2021).

To determine the inflammatory condition and investigate the therapeutic effects of MMP-responsive biomaterials, fluorescence imaging was considered for diagnosis and therapy.

Inflamed cartilage is characterized by MMP-13 overexpression and an acidic microenvironment. Therefore, MMP-13/pH-responsive ferritin nanocages (CMFn) loaded with an anti-inflammatory drug (hydroxychloroquine, HCQ), termed CMFn@HCQ, were constructed for OA imaging and therapy. CMFn is a marker for imaging diagnosis that emits light in response to MMP-13 overexpression. The intensity of CMFn light increases with the severity of OA. However, in normal joints, this compound emits no light. The release of HCQ causes an anti-inflammatory effect in OA joints to reduce synovial inflammation, and the retention time lasts up to 14 days (Chen et al., 2019a).

**FIGURE 2:**
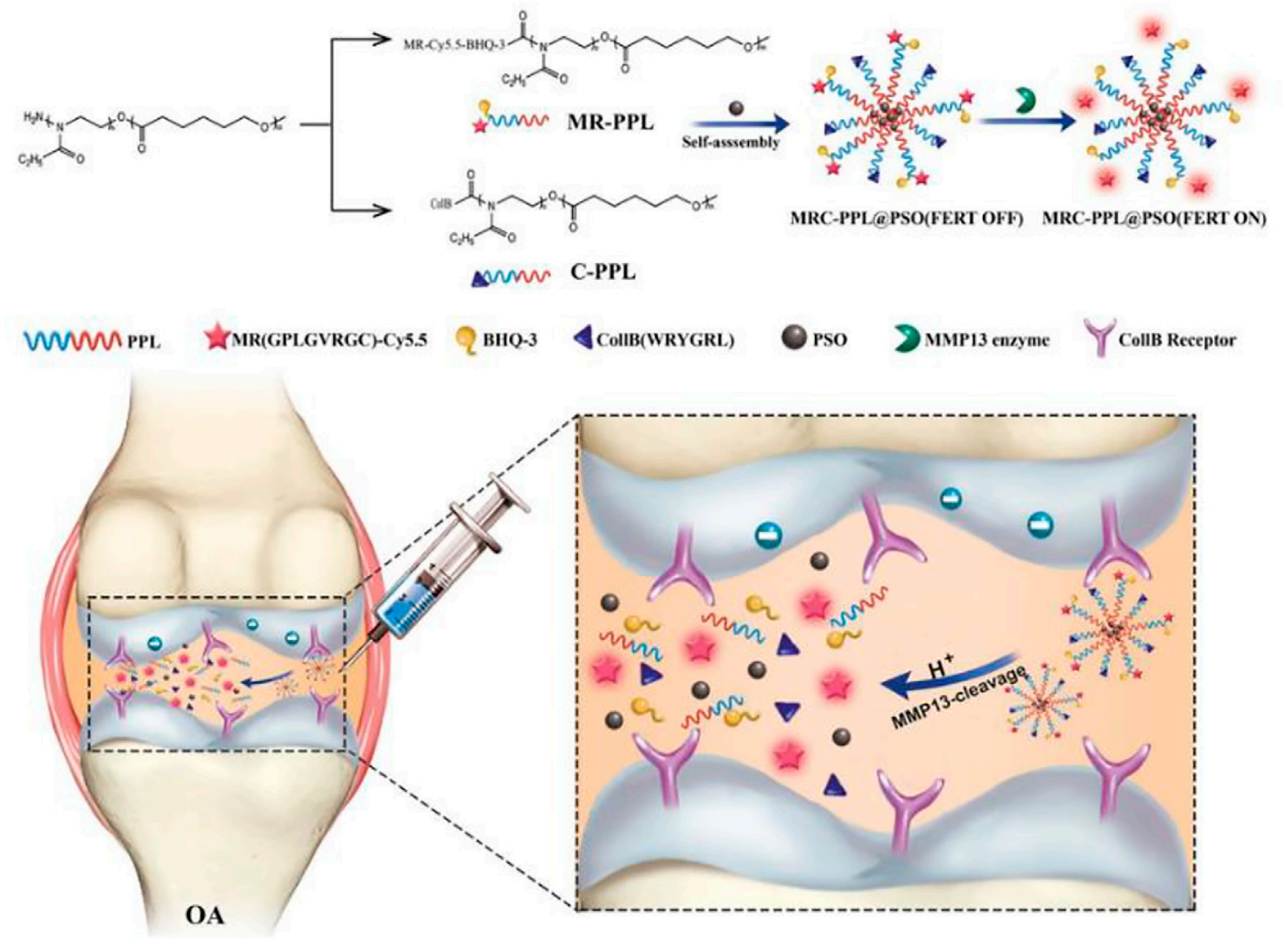
Schematic illustration of the synthesis and working mechanism of MMP-13 and pH responsive theranostic MRC-PPL@PSO nano-micelles for OA (Lan et al., 2020). Copyright, 2020, BMC.

Cartilage-targeting C-PPL was created by grafting collagen type II-targeting peptides with the sequence WRYGRL onto the polymer poly (2-ethyl-2-oxazoline)-poly (ε-caprolactone) (PPL). Additionally, PPL was conjugated with a specific peptide substrate of the MMP-13 enzyme (H2N–GPLGVRGC–SH) that was labelled with a fluorescent dye (Cy5.5) and was subsequently coupled with the black hole quencher-3 (BHQ-3) that can quench Cy5.5 fluorescence to obtain an MMP-13-responsive and pH-sensitive polymer (MR-Cy5.5-BHQ-3-PPL). A cartilage-targeting and OA-specific theragnostic nanoplatform (MRC-PPL) was obtained by the self-assembly of C-PPL and MR-PPL. Finally, MRC-PPL was loaded with the traditional Chinese medicine psoralidin (PSO) to form MRC-PPL@PSO nano-micelles, which specifically target and protect cartilage (Lan et al., 2020). The synthesis and mechanism of MRC-PPL@PSO nano-micelles to treat OA are shown in Figure 2.

In addition to MMP overexpression in arthritis tissue, the intrinsic properties of the OA microenvironment, especially synovial fluid, are also considered when designing novel nanomaterials. The increased activity of the GR enzyme was reported in the synovial fluid of RA and OA patients, and a selectively controlled drug release that is sensitive to the GR enzyme was designed for the treatment of arthritic diseases (Ostalowska et al., 2006; Sredzińska et al., 2009). Polymeric micelles were made of methoxypolyethylene glycol amine-glutathione-palmitic acid (mPEG-GSHn-PA) polymers. Dex was loaded into the cores of the polymeric micelles. The release of Dex was slow under physiological conditions, while the presence of the GR enzyme stimulated a burst release via a thiol−disulfide exchange between GSH and GSSG (Lima et al., 2021). The above biomaterials associated with MMPs are listed in Table 1.

**TABLE 1:** The biomaterials that target the upregulated enzymes in arthritis.

Arthritis	Enzymes-responsive group	Nanomaterial	Platform	Components	Responsive enzymes	Drug	Ref
RA	TGMS	Dex-loaded TGMS/DSPE-PEG2000	NPs	1.TGMS	MMP-3	Dex	He et al. (2020)
				2.Dex	MMP-9		
				3.PEG2000			
				4.DSPE			
RA	TGMS	TA-loaded TG-18 hydrogel	hydrogel	1.TGMS	MMP-2	TA	Joshi et al. (2018)
				2.TA	MMP-3		
					MMP-9		
RA	PEG	CEL-PRNPs	NPs	1.celastrol (CEL)	MMP-9		Deng et al. (2021)
				2.RGD			
				3.PEG			
				4.PLGA			
OA	H2N–GPLGVRGC–SH	CMFn@HCQ	nanocages	1.MMP-13 cleavble peptide	MMP-13	HCQ	Chen et al. (2019a)
				2.HCQ			
				3.collagen type II targeting peptides			
				4.BHQ3			
				5.Cy5.5			
				6.ferrritin			
OA	H2N–GPLGVRGC–SH	MRC-PPL@PSO	micelles	1.MMP-13 cleavble peptide	MMP-13	PSO	Lan et al. (2020)
				2.PSO			
				3.collagen type II targeting peptides			
				4.PPL			
				5.Cy5.5			
				6.BHQ-3			
OA	GSSG	Dex-loaded mPEG-GSHn-PA	micelles	1.PEG	GR enzyme	Dex	Lima et al. (2021)
				2.GSH			
				3.PA			
				4.Dex			

#### 2 Nanomaterials associated with downregulated enzymes in arthritis treatments

Apart from the destruction of cartilage tissue induced by the increased expression of MMPs, the decreased expression of oxide reductase associated with ROS showed a similar effect on cartilage. To reduce the expression of oxide reductase, the strategy was to supply these enzymes directly or mimic the activities with special biomaterials.

Supplementation with antioxidant enzymes such as SOD has been shown to be effective in treating arthritis. Chitosan was chemically conjugated with SOD to generate the nanoparticle-like conjugate 6-O-2′-hydroxylpropyltrimethyl ammonium chloride chitosan-SOD (O-HTCC-SOD), which was superior to unmodified SOD in bioavailability, prolonged half-life and residence in the rat joint cavity. After IA injection of O-HTCC-SOD into rats with MIA-induced OA, mechanical allodynia was greatly reduced, and changes in the gross morphological and histological lesions of articular cartilage were dramatically inhibited (Wang et al., 2020b).

Although the nanosized conjugate O-HTCC–SOD has exhibited higher enzyme activity and superior membrane permeability to native SOD, natural enzymes are unstable, expensive and difficult to store. Currently, biomaterials called nanozymes have been designed to mimic the effects of these oxide reductases. Nanozymes are a specific kind of nanomaterial that have the activities of intrinsic enzymes and possess unique advantages, such as high efficiency, increased compatibility with specific environments, such as high temperatures and pH variations, cyclic use, and a large surface area, and these materials can be conjugated to multiple ligands to achieve multifunctionality. These features give rise to their promising applications in a variety of fields (Pirmohamed et al., 2010).

Recently, numerous nanomaterials with enzyme-like properties have been discovered for OA treatment, including metals, metal oxides, and carbon-based materials.

As a representative metal oxide, cerium oxide has been evaluated in RA treatment. Engineered cerium oxide (CeO_2_) nanoparticles (CeONPs), which are also known as nanoceria, have attracted much attention for exhibiting SOD^−^, CAT^−^, and oxidase-like activity (Heckert et al., 2008; Baldim et al., 2018; Kalashnikova et al., 2020). In reduction reactions, SOD catalyses O_2_^•−^ into H_2_O_2_, which may undergo catalysis by CAT into H_2_O.

Given that albumin is a natural protein and scavenging receptors are widely distributed in the inflamed joints of RA, albumin-nanoceria NPs (A-nanoceria) were synthesized by connecting albumin to nanoceria and further conjugated with near-infrared, indocyanine green (ICG) dye. Enzymatic properties and ROS scavenging activities against a monocyte cell line and systemic targeting potential were evaluated in a collagen-induced arthritis (CIA) mouse model. Such a design has the advantages of targeting inflammation, assessing severity, and controlling inflammation with imaging guidance in RA (Kratschmer et al., 1990).

Moreover, carbon-based materials have also exhibited the activities of nanozymes in scavenging ROS. Fullerene (C60) is a spherical carbon molecule with a unique cage structure that functions as a free-radical scavenger. Apart from inhibiting ROS-induced catabolism in cartilage, fullerene also decreases friction on the cartilage surface and subsequently prevents the further development of cartilage degeneration. With these advantages, fullerene has been used to synthesize biomaterials for the treatment of arthritis. For example, fullerene-like MoS_2_ (F-MoS_2_) NPs are efficient lubricants and antioxidants for artificial synovial fluid. These NPs possess intrinsic dual-enzyme-like activity, mimicking SOD and CAT under physiological conditions (pH 7.4, 25°C) and regulating the ROS level in artificial synovial fluid containing HA (Chen et al., 2019b).

Prussian blue (PB) has been approved by the U.S. FDA as a commonly used dye and medicine due to its excellent biocompatibility and biosafety. The peroxidase, CAT, and SOD activities of PBzymes mediate the scavenging of •OH, •OOH, and H_2_O_2_, exhibiting outstanding anti-inflammatory and antioxidative bioactivities (Long et al., 2016; Zhang et al., 2016; Dacarro et al., 2018; Qin et al., 2018).

A hollow PBzyme (HPBzyme) with a mesopore structure and a high specific surface area was produced that could remodel the OA microenvironment by mitigating the inflammatory response, protecting against chondrocyte ECM degradation, and exhibiting therapeutic efficacy *in vivo* (Hou et al., 2021).

PB has also been integrated into other therapeutic approaches, such as exosomes and ultrasound, for arthritis treatment. Low-density ultrasound is a noninvasive biophysical treatment that can reduce joint swelling and inflammation in OA models (Iwabuchi et al., 2014). The combined therapeutic effects of PB and low-density ultrasound on animal OA by scavenging oxygen free radicals was investigated. It was found that this treatment could significantly remove ROS, alleviate ROS-induced apoptosis, and reduce the degeneration of articular cartilage (Zuo et al., 2021). Furthermore, neutrophil-derived exosomes engineered with ultrasmall PB nanoparticles (uPB-Exo) have been shown to be effective in treating RA. uPB-Exo selectively accumulated in activated fibroblast-like synoviocytes and acted as mimics of SOD2 and NOX2 in inflamed joints of RA *in vivo*, subsequently neutralizing proinflammatory factors, alleviating inflammatory synovitis and protecting against cartilage damage in an advanced RA mouse model (Zhang et al., 2022).

Selenium (Se) is an essential dietary nutrient and has been reported to have lower serum concentrations in RA patients than healthy individuals (Yu et al., 2016). Supplementation with Se is controversial in the treatment of arthritis is controversial due to its toxicity. Nanosized Se is known to have superior antioxidant effects and reduced toxicity (Malhotra et al., 2016). In a rat RA model, SeNPs exhibited potent anti-inflammatory effects and promoted the expression of CAT, SOD and GPX (Ren et al., 2019b).

**FIGURE 3:**
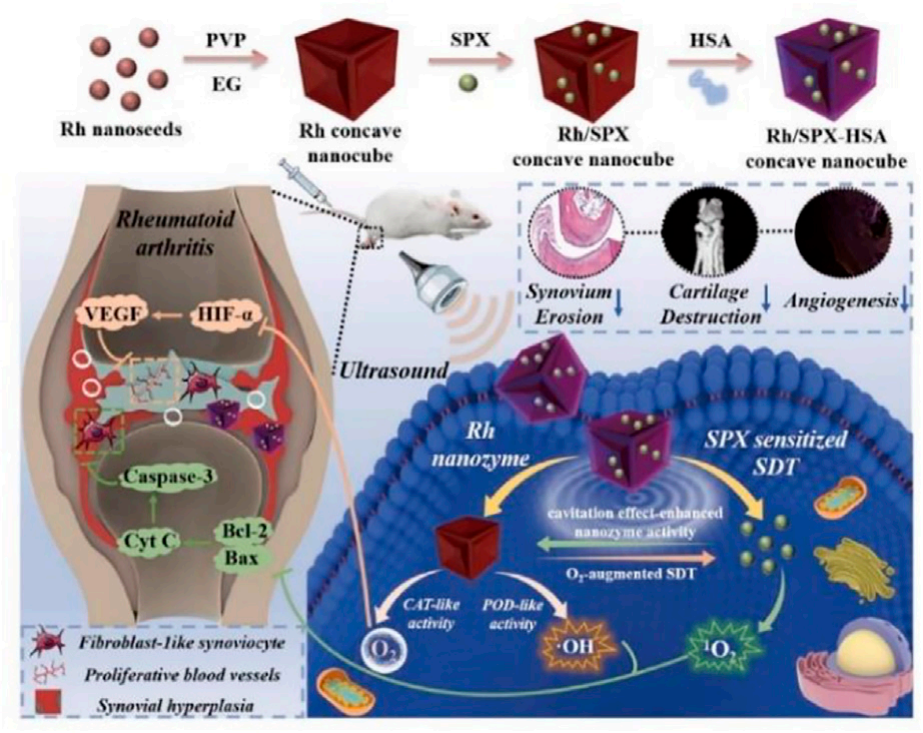
Schematic illustration of the synthesis and working mechanism of MMP-13 and pH responsive theranostic MRC-PPL@PSO nano-micelles for OA (Li et al., 2021). Copyright, 2021, Elsevier.

Ultrasound, which is a noninvasive biophysical therapy and a common mode of sonodynamic therapy (SDT), can strongly penetrate inflammatory tissues and kill inflammatory cells, thus reducing synovial hyperplasia and minimizing oxidative damage to surrounding normal tissues. SDT is hampered by the hypoxic microenvironment of RA caused by fibroblast-like synoviocyte (FLS) proliferation. Rhodium NP (Rh) nanozymes with concave-cube shapes could compensate for the deficiency of ultrasound therapy by exhibiting the activities of POD and CAT, which generate O_2_ and •OH to alleviate hypoxia. In addition to its remarkable sonosensitive properties, the antibacterial drug sparfloxacin (SPX) can reside for a long time in joint tissues after systemic administration, which makes it possible to target the abnormal proliferation of FLSs in synovial tissue in the joint and block the development of RA. A small glycoprotein rich in cysteine known as SPARC is overexpressed in the synovial fluid and synovium from RA patients and increased in mice with CIA (Liu et al., 2019). SPARC has high affinity for human serum albumin (HAS) (Park et al., 2019). Therefore, HSA-modified Rh/SPX nanozyme was fabricated for RA treatment by combining the advantages and characteristics of these components (Li et al., 2021). The preparation of Rh-SPX/HSA and its related mechanisms in the treatment of RA are shown in Figure 3.

In addition to the combination of ultrasound and nanozymes to treat arthritis, a promising technique that combines near-infrared (NIR) with nanozymes for the treatment of OA was proposed. Epigallocatechin gallate (EGCG)-coated Au-Ag nanojars (E@Au-Ag) were produced based on the POD-like activity of Au-Ag and the scavenging of oxygen free radicals by EGCG, which is sensitive to NIR. These multifunctional enzyme-like nanomaterials can repair mitochondrial damage, promote cartilage migration, and reduce chondrocyte apoptosis (Xu et al., 2022). Biomaterials associated with antioxidant enzymes for arthritis treatment are listed in Table 2.

**TABLE 2:** Biomaterials that mimic the downregulated enzymes in arthritis.

Arthritis	Nanomaterial	Platform	Components	Enzyme mimics	Drug	Ref
RA	A-nanoceria-ICG	NPs	1.Albumin	SOD	metal oxides	Kratschmer et al. (1990)
			2.cerium oxide	CAT		
			3.ICG	POD		
OA	F-MoS_2_	NPs	1.Fullerene	SOD	carbon-based materials	Chen et al. (2019b)
			2.MoS2	CAT		
OA	HPBzyme	NPs	Prussian blue	POD	Prussian blue	Hou et al. (2021)
				CAT		
				SOD		
OA	PBNPs	NPs	Prussian blue	POD	Prussian blue	Zuo et al. (2021)
				CAT		
				SOD		
RA	uPB-Exo	NPs	1.neutrophil-derived exosomes	SOD2	Prussian blue	Zhang et al. (2022)
			2.Prussian blue	NOX2		
RA	SeNPs	NPs	Selenium	SOD	metal	Ren et al. (2019b)
				CAT		
				GPx1		
RA	HSA-modified Rh/SPX	nanocube	1.human serum albumin (HSA)	POD	noble metal	Li et al. (2021)
			2.Sparfloxacin (SPX)	CAT		
			3.Rhodium (Rh)			
OA	E@Au-Ag	nano-jars	1.EGCG	POD	noble metal	Xu et al. (2022)
			2.Au-Ag			

## Discussion

OA and RA are both inflammatory diseases. RA is a systemic disease that affects joints all over the body, especially the overloaded knee joints, and affects normal movement (Radu and Bungau, 2021). OA is a local joint disease, which is common in patients with metabolic syndrome, trauma, and aging (Whittaker et al., 2021). In comparing OA and RA, a striking similarity in gene expression is found. For example, the increased levels of MMPs and the decreased expression of antioxidant enzymes occur in OA and RA, but the differences also exist. MMP-9 is the main enzyme that causes RA while MMP-13 is reported to be the most important enzyme for the development of OA. Meanwhile, in terms of pathological changes, the proliferation of synovial tissue and blood vessels in RA was more obvious than that in OA. Macrophages distributed in synovial tissue and osteoclasts from subchondral bone were the main sources of inflammation, ultimately leading to the destruction of cartilage. Therefore, chondrocytes, osteoclasts and macrophages have been the main targets for arthritis treatment with different biomaterials.

RA is a systemic inflammatory disease, and joint destruction is generally more intense than that in OA. Compared to IA injection, oral drug delivery for arthritis causes severe side effects. Recently, pain has been primarily controlled with corticosteroids and hyaluronic acid via IA injection. It is possible to deliver high drug concentrations directly to osteoarthritic joints through direct IA delivery. The administration of IA corticosteroids efficiently reduces articular pain and synovitis, but high concentrations of corticosteroids can also damage chondrocyte metabolism, causing changes in ECM composition and articular cartilage structure. A novel treatment for arthritis is urgently needed.

Enzymes are involved in various physiological reactions and participate in the proteolytic degradation of proteins and complex regulatory signalling pathways. Aberrant expression of these enzymes in the human body plays a critical role in pathological processes, especially inflammatory reactions. Different types of MMPs were upregulated by inflammatory factors and subsequently degrade the ECM. In addition to MMPs, ROS also participate in the development of arthritis. The generation of ROS is inhibited by endogenous antioxidants such as SOD, CAT, GPX, and heme oxygenase (HO-1). Despite the complex pathological process of arthritis, different types of arthritis including OA and RA share the common features: the increased levels of MMPs and the decreased expression of antioxidant enzymes. Hence, it is extremely feasible to design nanomaterials based on these enzymes as molecular targets for arthritis therapy.

Although nanomaterials have the advantages of high biocompatibility and bioavailability due to their structural and functional characteristics, the biosafety of nanomaterials cannot be ignored (Chen et al., 2021-09). Nanomaterials enter the body through ingestion, injection, inhalation and skin contact and subsequently accumulate in organs through blood flow, affecting the structure and function of organs (Ai et al., 2011-01). For arthritis treatment, intra-articular injection of enzyme-related biomaterials can guarantee the controlled release and targeted therapy without affecting other tissues or organs through blood circulation. Natural polymers are more suitable and safer for clinical application due to their biodegradation. Especially, hyaluronic acid from cartilage tissue has been commonly used for biomaterial. It is a promising strategy for arthritis treatment through discovering more biologically active materials from the human body in the future and combining them with drugs to regulate the expression of the enzymes mentioned above.

Given that cartilage and the synovium are affected in arthritis, various NPs that target these upregulated or downregulated enzymes mainly act on these sites, especially macrophages from the synovium and OCs from the subchondral bone. Both macrophages and osteoclasts are inflammatory cells with the same receptor on the surface of the membrane and release inflammatory factors. Therefore, biomaterials that target these inflammatory cells or chondrocytes are the current options for arthritis treatment. For the treatment of inflammatory arthritis, nano-drug delivery technologies that respond to subchondral enzymes are rare. There are technical challenges, such as how to penetrate the cartilage and reach the deep layer to target OCs that destroy the subchondral bone. Second, aside from MMPs and endogenous reductase, many enzymes are also involved in the pathological processes of arthritis. The expression of cyclooxygenase-2 (COX-2) in joints has also been linked to synovial inflammation in arthritis, and COX-2 inhibitors (celecoxib) have been frequently used and have shown therapeutic benefits in arthritis. Synergistic treatments targeting several enzymes may obtain better results. Finally, avoiding rapid clearance after IA injection is critical for maintaining drug concentrations and guaranteeing efficacy.

It should be noted that the current studies regarding enzyme-related biomaterials in the field of arthritis are not numerous; nanotherapies are extremely challenging and are also promising based on the molecular mechanism underlying arthritis.
